# Functional bias in molecular evolution rate of *Arabidopsis thaliana*

**DOI:** 10.1186/1471-2148-10-125

**Published:** 2010-05-01

**Authors:** Andrew S Warren, Ramu Anandakrishnan, Liqing Zhang

**Affiliations:** 1 Department of Computer Science, Virginia Tech, Blacksburg, VA, USA; 2 Program in Genetics, Bioinformatics, and Computational Biology, Virginia Tech, Blacksburg, VA, USA

## Abstract

**Background:**

Characteristics derived from mutation and other mechanisms that are advantageous for survival are often preserved during evolution by natural selection. Some genes are conserved in many organisms because they are responsible for fundamental biological function, others are conserved for their unique functional characteristics. Therefore one would expect the rate of molecular evolution for individual genes to be dependent on their biological function. Whether this expectation holds for genes duplicated by whole genome duplication is not known.

**Results:**

We empirically demonstrate here, using duplicated genes generated from the *Arabidopsis thaliana α*-duplication event, that the rate of molecular evolution of genes duplicated in this event depend on biological function. Using functional clustering based on gene ontology annotation of gene pairs, we show that some duplicated genes, such as defense response genes, are under weaker purifying selection or under stronger diversifying selection than other duplicated genes, such as protein translation genes, as measured by the ratio of nonsynonymous to synonymous divergence (*dN*/*dS*).

**Conclusions:**

These results provide empirical evidence indicating that molecular evolution rate for genes duplicated in whole genome duplication, as measured by *dN*/*dS*, may depend on biological function, which we characterize using gene ontology annotation. Furthermore, the general approach used here provides a framework for comparative analysis of molecular evolution rate for genes based on their biological function.

## Background

Gene duplication has been considered to be an important process for creating novel gene function [1,2]. Duplicated genes give organisms the opportunity to "experiment" with mutations without losing the biological function of the original gene because when one copy experiences a deleterious mutation that destroys its function, the other copy can still be functional. Beneficial mutations can be retained due to the advantages they confer. Neutral mutations, such as most synonymous mutations, or some nonsynonymous mutations that do not alter gene function, can also be retained. With accumulated mutations, the pair of duplicate genes will diverge in sequence, and possibly function, over time. The amount of sequence divergence depends on (a) the natural mutation rate, determined by environment condition, error correction mechanism, GC content etc., and (b) the selective constraint due to a gene's biological function, i.e. biological functions that are more likely to benefit from mutations could be under diversifying selection whereas those that are less likely to benefit from mutations could be under purifying selection [3-10].

Gene sequence divergence can be measured by the number of nonsynonymous mutations per site, *dN*, i.e nucleotide substitutions that change the amino acid encoded by the codon. However, in addition to selection due to evolutionary pressure, other factors such as expression level [11], guanine-cytosine (GC) content [12], and location on the chromosome [13], have been found to affect *dN*. To isolate the effect of selective constraint, *dN *can be compared to the number of synonymous mutations per synonymous site, *dS*, i.e. nucleotide substitutions that *do not *change the amino acid encoded by a given codon. *dS *is also affected by expression level, GC content [14,15], and chromosomal location [16]. Because synonymous changes do not change the amino acid, they are often considered to be selectively neutral (although evidence shows that synonymous changes can be weakly selected [15]). Thus the ratio of nonsynonymous to synonymous mutations, *dN*/*dS*, should measure the net effect of selection on molecular evolution rate, assuming the other factors equally affect synonymous and nonsynonymous mutations. A study by Williams and Hurst [13] indicates that in some cases chromosome location may not equally affect *dN *and *dS*.

Drummond et al. [11] indicated that expression level has a larger effect on *dN *compared to *dS*. Duret et al. [17] found that substitution rates differ significantly by tissue in mammals.

Previous work in plants reveal that rates of nonsynonymous substitutions were negatively correlated with GC content at synonymous third codon positions, and synonymous substitution rates were negatively correlated with codon bias, similar to what has been found in the animal system [18]. Moreover, a study of 83 genes in *Arabidopsis thaliana *and *Arabidopsis lyrata *has shown a significant negative correlation between the rates of nonsynonymous substitutions and gene expression level [15]. Ganko et al. [19] found that average expression level and breadth of expression tend to decline with *dN *in duplicated genes of Arabidopsis but do not have a significant correlation with *dS*. Furthermore, it has been well documented that some functional classes of genes have distinct expression levels [20]. However, there has not been a large-scale study on how variations in selective constraint are reflected in gene functions. In this work we use the genes from the *Arabidopsis thaliana *gene duplication events of 20-60 MYA [21,22], i.e. the *α*-duplication event, as a benchmark to investigate the effect of biological function on molecular evolution rate of the duplicated genes. Using a single genome duplication event eliminates variations in divergence rates due to factors such as population size that may be different for different organisms.

A whole genome duplication event in an ancestral species creates many paralogs. Some of these genes may be discarded over time. There is evidence for a functional bias for those paralogs that are retained [6]. Those that are retained tend to be functionally redundant and take advantage of the dosage effect or have functional divergence through neo- and subfunctionalization. However, "the incidence of functional divergence among duplicated genes is difficult to quantify" [23]. The objective of this study is to empirically examine the relationship between biological function and the rate of sequence divergence after a duplication event. To this end we have employed Gene Ontology (GO) annotations in our analysis to describe the common biological role of a paralogous pair. Because many of the duplicated genes are also experimentally characterized in the TAIR gene ontology (GO) dataset [24], it may be possible to discern properties of evolutionary rates based on GO categories.

## Methods

We used the Bowers et al. [22] dataset for the *A. thaliana *whole genome *α*-duplication event, which based on estimation, occurred before the divergence of *Arabidopsis thaliana *from Brassica but after its divergence from the Malvaceae.

**Figure 1:**
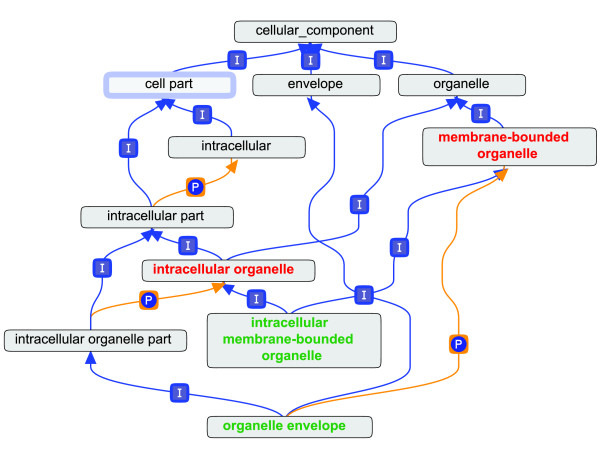
**Shared Function Combination**. The following example demonstrates how GO annotations for two genes in a pair might be combined. If gene1 has GO function of organelle envelope, and gene2 intracellular membrane-bound organelle (green terms), their shared functions would be membrane-bound organelle and intracellular organelle (red terms). Edges marked with 'P' and 'I' stand represent the "part of" and "is a" arcs of GO respectively.

To ensure the quality of the analysis we screened GO annotations from the TAIR dataset based on their evidence codes. We removed those pairs from the analysis set if at least one gene in a pair did not have an annotation that was curated or experimentally assigned. If either gene in a gene pair was not annotated experimentally or by curator, the pair was excluded from the analysis. In order to make a direct link between function and the molecular evolution rate we labeled each pair with only their most specific shared functions from GO terms. We also required that these shared functions were at a depth of one or greater (assuming the nodes "Biological Process", "Cellular Component", and "Molecular Function" have a depth of zero). An example is shown in Figure 1. Here we define the depth of a term to be the minimum distance over all paths from the root to that term's node. GO annotations were obtained from TAIR on June 4, 2009.

To assess the overall effect of gene function on sequence divergence, we placed genes into functional groups. The objective in creating these groups can be defined as follows. For any two gene pairs the more specific their shared role in the cell the tighter their subsequent grouping. To do this we used the GOSim package [25] to cluster genes based on their functional profile. The GOSim package "provides the researcher with various information theoretic similarity concepts for GO terms." Within the GOSim package we selected the Resnik method [26] to create term, term similarities for all pairs of terms in the Gene Ontology. This is defined to be: , where *Pa*(*t, t'*) denotes the term set of all common ancestors of GO terms *t *and *t'*, and *IC*(*t*) is the information content of term *t *as defined by Lord et al. [27]. These similarities were combined using the "optimal assignment" method by Frohlich et al. [28] to give coefficients, for all pairwise combinations of gene pairs, that indicate the functional similarity of selected pairs. The optimal assignment method assigns each term of the gene with fewer annotations to exactly one term of the other gene, such that the sum of term-term similarities is maximized. Based on these coefficients, Ward's hierarchical clustering algorithm [29] was used to group genes together with similar functional profiles. The resulting hierarchical tree was cut using a bottom up approach such that each group meets a minimum size constraint of ≥ 20. In order to maintain good functional specificity for shared functions, without reducing the population of the resulting groups to a trivial number, groups were defined by the lowest internal node that achieved the minimum size threshold. For Ward's clustering algorithm the height corresponds to the analysis of variance (ANOVA) sum of squares difference between two clusters added up over all the variables within those clusters.

The protein and DNA sequences were obtained from The Arabidopsis Information Resource (TAIR) database on February 20, 2008 [30]. We aligned the protein sequences for duplicate gene pairs using the *needle *program, with default parameters. The program implements the Needleman-Wunsch global alignment algorithm [31]. We then aligned the DNA sequences for duplicated gene pairs according to the aligned protein sequences using the *PAL*2*NAL *program [32]. Last, to calculate *dN*/*dS *for duplicate gene pairs, we use the *yn*00 with default parameters within the Phylogenetic Analysis of Maximum Likelihood (PAML) program [33]. *yn*00 implements the method of Yang and Nielsen [34] which calculates *dN*/*dS *taking into account transition/transversion rate biases and base/codon frequency biases.

To evaluate statistical correlation between *dN*/*dS *and functional groups we used the analysis of variance (ANOVA) method [35]. ANOVA uses Fisher's F-test to determine statistical significance of variance in group means compared to the mean for a group. The resulting *p*-value determines if the null hypotheses, that mean *dN*/*dS *values are equivalent for all functional groups, should be rejected. To identify the specific groups that are significantly different, we used Tukey's honestly significant difference (HSD) criteria which is based on Studentized range distribution for determining critical values [36].

To characterize specific functional groups we examine the group's relative enrichment of GO annotation for genes in the group compared to all the genes in the *α*-duplication event. For this purpose we use the Ontologizer software's parent-child union method with Bonferroni correction [37].

## Results

There are a total of 3822 duplicated gene pairs from the *α *duplication event. After removing the gene pairs that only have electronic annotation of GO terms, we had 2728 duplicated gene pairs. Then after removing the gene pairs that have shared gene function that is at depth 1, we were finally left with 2683 duplicated gene pairs.

**Figure 2:**
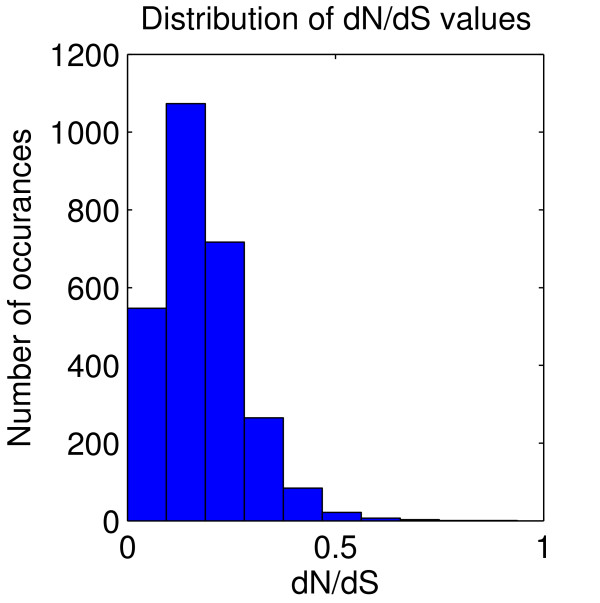
**DNDS Distribution**. Distribution of *dN*/*dS *values for all gene pairs in the *α *duplication event. Values are grouped into bins of size 0.1.

The distribution of *dN*/*dS *values for the 2728 duplicated gene pairs is shown in Figure 2. The *dN*/*dS *values range from 0 to 0.8, but most of the gene pairs have *dN*/*dS *values less than 0.5, and 86% have *dN*/*dS *< 0.3, suggesting that most of the duplicated gene pairs were under purifying selection. No gene pairs have *dN*/*dS *> 1, thus there is no indication of strong positive selection in these recently duplicated genes.

The gene pairs were separated into functional groups using the clustering method as described in the Methods section above. All gene pairs were grouped based on each of the three GO categories resulting in three sets of groups: 40 groups for biological process, 48 groups for molecular function, and 26 groups for cellular component. The analysis of variance method was then used to determine if there is a dependence between *dN*/*dS *and functional groups of gene pairs. Results of the analysis, which are summarized in Table 1, show a *p*-value < 10^-16 ^for all three groupings.

**Table 1:** ANOVA Groups.

Source	SS	df	MS	F	Prob > F
Biological Process					

Groups	2.5427	39	0.652	8.36	< 10^-16^
Error	10.5929	1358	0.0078		
Total	13.1356	1397			

Molecular Function					

Groups	3.5768	47	0.0761	9.14	< 10^-16^
Error	16.5045	1982	0.00833		
Total	20.0813	2029			

Cellular Function					

Groups	2.4666	25	0.09866	10.1	< 10^-16^
Error	15.9469	1633	0.00977		
Total	18.4135	1658			

Analysis of Variance for *dN*/*dS *by functional group

**Figure 3:**
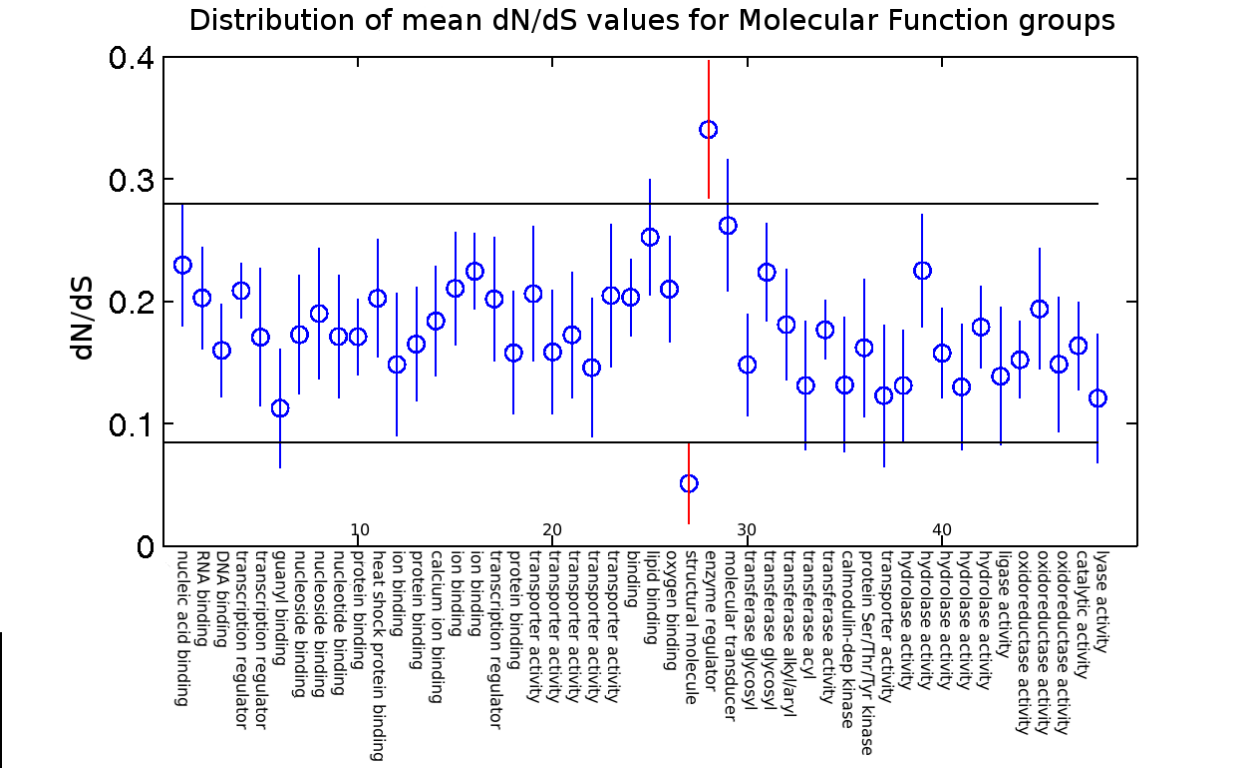
**Molecular Function Means**. Tukey's HSD test for *dN*/*dS *as a function of functional grouping based on molecular function (MF). A list of all the groups is included in the supplementary material along with the name of the top three GO terms representing each group. The 95% confidence intervals for group means are indicated by vertical lines. The horizontal lines are used to visually identify groups with extreme mean *dN*/*dS *values (red vertical lines). Results show that genes representing "enzyme inhibitor activity" (group 28) have significantly higher *dN*/*dS *values on average, while "structural component of ribosome" (group 27) have significantly lower *dN*/*dS *values.

**Figure 4:**
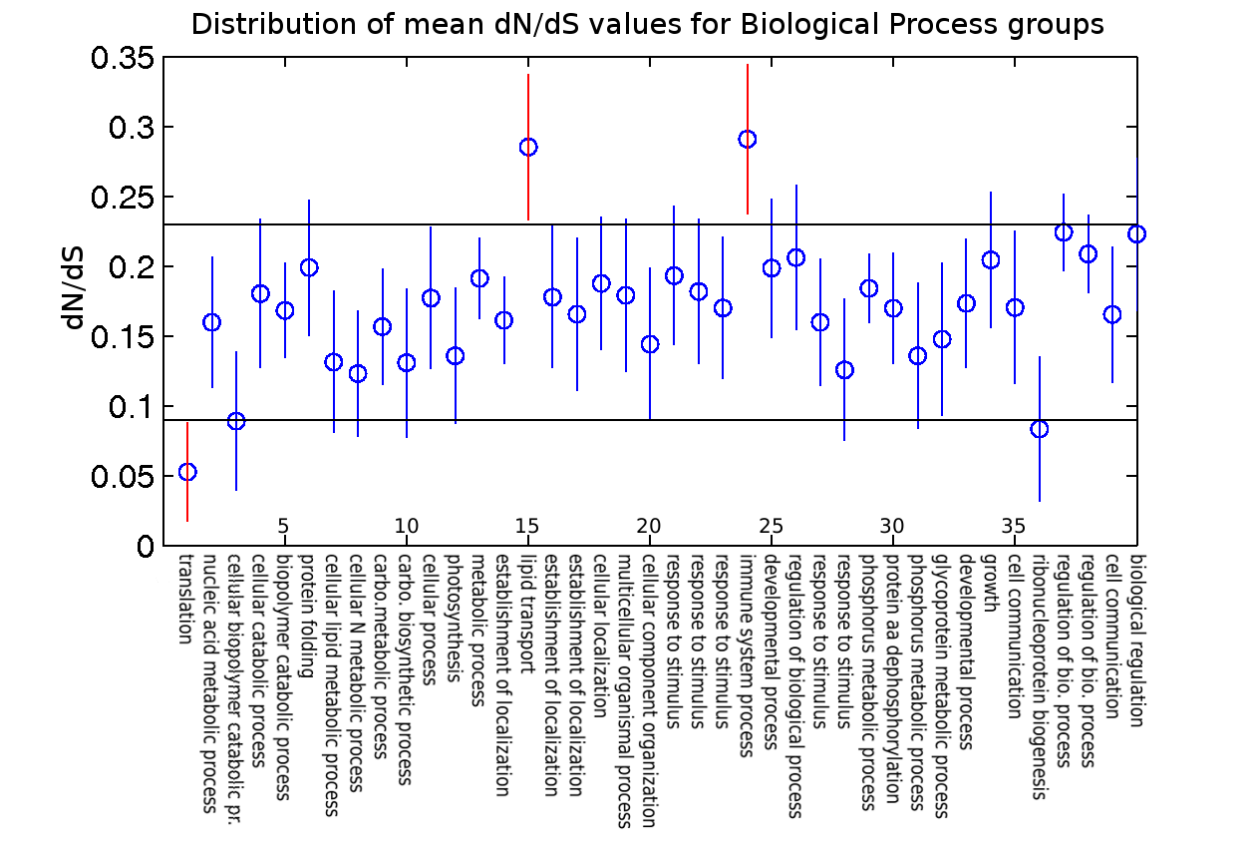
**Biological Process Means**. Tukey's HSD test for *dN*/*dS *as a function of functional grouping based on biological process (BP). A list of all the groups is included in the supplementary material along with the name of the top three GO terms representing each group. The 95% confidence intervals for group means are indicated by vertical lines. The horizontal lines are used to visually identify groups with extreme mean *dN*/*dS *values (red vertical lines). Results show that genes representing "lipid transport" (group 15), and "defense response" (group 24) have significantly higher *dN*/*dS *values on average, while "cellular macromolecule biosynthesis" (group 1) have significantly lower *dN*/*dS *values.

**Figure 5:**
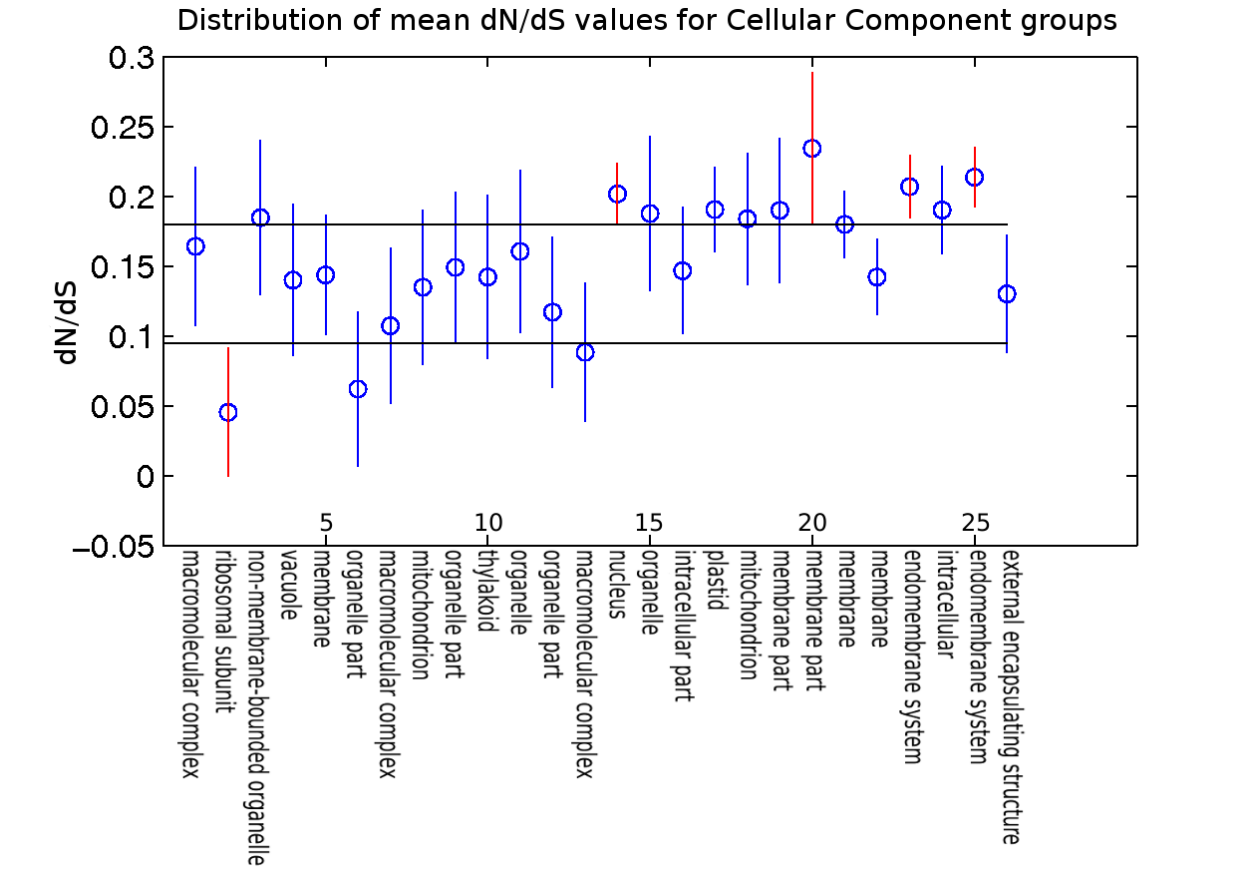
**Cellular Component Means**. Tukey's HSD test for *dN*/*dS *as a function of functional grouping based on cellular component. A list of all the groups is included in the supplementary material along with the name of the top three GO terms representing each group. The 95% confidence intervals for group means are indicated by vertical lines. The horizontal lines are used to visually identify groups with extreme mean *dN*/*dS *values (red vertical lines). Results show that "anchored to membrane" (group 20), "nucleus" (group 14), "endomembrane system"(group 23 & 25) have significantly higher *dN*/*dS *values on average, while "cytosolic ribosome" (group 2) genes have significantly lower *dN*/*dS *values.

This indicated a strong difference between mean *dN*/*dS *for groups based on biological function as represented by the three GO categories. Figure 3, Figure 4, and Figure 5 show the groups of duplicated gene pairs, their mean *dN*/*dS *values and the 95% confidence intervals for the means based on Tukey's HSD test. There are differences between the distribution of *dN*/*dS *values across different groups. The groups that are most different for each of the three sets of groupings, biological process, molecular function and cellular process are highlighted in the figures.

We performed functional enrichment analysis to identify the primary biological function (GO term) representing each of the groups. The top three GO terms from the enrichment analysis for all the groups and visualizations of enrichments for the examples mentioned here are provided in Additional file 1. Figure 3 shows that molecular function group 27 and group 28 genes have a sharp contrast in *dN*/*dS *values. Group 27 is enriched with genes that are structural constituent of ribosome (GO:0003735, *p*-value = 2.11E-240). In contrast, group 28 is enriched with genes that have the molecular function of, enzyme inhibitor activity (GO:0004857, *p*-value = 1.44E-079) and pectinesterase inhibitor activity (GO:0046910, *p*-value = 3.42E-074). We noticed that group 29 also has a relatively high mean *dN*/*dS*, and is enriched with genes with signal transducer activity (GO:0060089, *p*-value = 1.72E-039) and activation of innate immune response (GO:0002218, *p*-value = 1.26E-005), which seems to be consistent with the fact that immune related genes tend to evolve fast. Group 25 also has elevated *dN*/*dS *values and is enriched for lipid binding (GO:0008289, *p*-value = 1.35E-078). An ontology enrichment figure for these groups can be found in Additional file 2.

Figure 4 shows that groups 1, 15 and 24 genes have a sharp contrast in *dN*/*dS *values. Group 1's slowly-evolving genes are enriched with functions involved in the cellular macromolecule biosynthetic process (GO:0034645, *p*-value = 1.70E-078). In contrast, group 15 has elevated *dN*/*dS *values and is enriched with genes involved in lipid transport (GO:0006869, *p*-value = 1.62E-093). All but one pair of genes from this group are members of Group 25 (enriched for lipid binding) from the molecular function analysis. The other group with high *dN*/*dS *values, Group 24, has genes involved in defense response (GO:0006952, *p*-value = 6.48E-047), response to stimulus (GO:0050896, *p*-value = 3.11E-022), and activation of immune response (GO:0002253, *p*-value = 7.31E-004). An ontology enrichment figure for these groups can be found in Additional file 3.

Figure 5 shows that group 2 and group 20 genes have a sharp contrast in *dN*/*dS *values. Analysis of functional enrichment in these groups shows that group 2 is enriched with genes that make up components of cytosolic ribosomes (GO:0022626, *p*-value = 1.61E-115). Group 20 is enriched with genes whose protein products are anchored to membrane (GO:0031225, *p*-value = 7.67E-94). Group 14 has a high *dN*/*dS *value and is significantly enriched for genes whose products are contained in the nucleus (GO:0005634, *p*-value = 1.32E-107). Further analysis of this group with respect to molecular function shows enrichment for genes involved in transcription regulator activity (GO:0030528, *p*-value = 2.53E-265). Two other groups with genes having high *dN*/*dS*, groups 23 and 25, are enriched with genes that are components of endomembrane system. Out of the 872 genes found to be annoated with endomembrane system in the *α*-duplicated genes, 623 were found to be in groups 23 and 25. An ontology enrichment figure for these groups can be found in Additional file 4.

## Discussion

When making predictions about evolution, a frequent caveat is that the results are given under the assumption that the genes/proteins involved evolve at some constant rate defined as a parameter in the model. Bos and Pasada [38] have suggested fitting the model to the genes being used to obtain more accurate results. Why not then also adjust the rate at which the genes are assumed to evolve based on selective pressure? We are not aware of any data that exists which defines, in broad fashion, the tendency of genes to diverge at a certain rate based on its functional characteristics. Perhaps the most similar study to that done here, Blanc and Wolfe [23] investigate a similar but distinct issue by comparing the divergence of paralogous pairs to an outgroup protein. They found a statistically significant divergence rate for 173 out of the 833 paralogous pairs analyzed. However, their analysis was conditioned on the choice of an appropriate outgroup protein. They investigated functional enrichment for duplicated pairs where one gene diverges faster than the other in comparison to an outgroup protein, i.e., "asymmetric divergence". In their work the aim is to evaluate the probability that "the two duplicated protein sequences evolve at the same rate." In this work we are looking for significant differences in the average *dN*/*dS *ratio for groups that have been clustered according to their functional profiles and use only the differences between two *α*-duplicated genes.

The analysis we employ presents compelling evidence for functional bias in molecular evolution rate of duplicated genes arising from the Arabidopsis *α *duplication event. However, it is possible that there is another cause for the differences in *dN*/*dS*, or that some of the conditions imposed in our analysis has inadvertently biased the results. To try and account for some of these possibilities we examine the effect of chromosome location, combining multiple and single duplicates, and the clustering parameters for defining functional groups.

### Effect of chromosomal location

A recent study has shown that genes from the same functional classes tend to cluster together on the chromosomes in *Arabidopsis thaliana *[39]. Also, gene expression is highly correlated between neighboring genes on a chromosome in *Arabidopsis thaliana *[40]. In addition, the distribution of tandemly arrayed genes appears to be positively correlated with recombination rates in both *Arabidopsis thaliana *[41] and rice [42]. Here we examined whether the rate of evolution in duplicated genes generated from the *α *duplication event in Arabidopsis is a function of chromosome location. The chromosome location can be calculated as the distance, in base pairs (bp) from the centromere to the midpoint of the gene. The correlation coefficient between *dN*/*dS *and chromosome location is 0.006 (*p*-value = 0.7) indicating that for the dataset used here, distance from the centromere is not the cause of variations in *dN*/*dS *values. Chromosomal location of duplicated gene pairs are shown in Additional file 5.

### Effect of combining multiple and single duplicates

The *Arabidopsis thaliana α*-duplication dataset contains a number of genes that have more than one duplicate due to subsequent gene duplications. We refer to these genes as multiple duplicates and refer to the genes that do not have additional duplications following *α*-duplication as single duplicates. Analysis of variance between *dN*/*dS *and multiple/single duplicates has a *p*-value of < 10^-16 ^indicating that copy number does influence the rate of divergence for multiple/single duplicates. A two-way analysis of variance between *dN*/*dS *and both functional groups and multiple/single duplicates also has a *p*-value of < 10^-16 ^indicating that there is an interdependence between functional groups and multiple/single duplicates. Furthermore, an analysis of variance excluding genes with multiple duplicates does identify protein translation genes as having a significantly lower *dN*/*dS *value, but does *not *contain a sufficient number defense response genes to establish a functional group. Most (22 of the 27) defense response pairs have genes with multiple duplicates while few (7 of the 53) protein translation pairs have genes with multiple duplicates. This analysis shows that genes with multiple duplicates tend to diverge at a faster rate (i.e. high *dN*/*dS*) than other genes. A figure illustrating this difference can be found in Additional file 6. As there is no reason to believe that some genes duplicate more frequently than others, the duplicates for defense response genes must be retained more frequently than others. This is what one would expect if there were weaker negative selection against mutations in the defense response genes, stronger selective pressure and constraints on mutations in protein translation genes. This is further evidence that natural selection favors more variation in defense response genes, while variation in translation genes are selected against, probably to maintain this fundamental biological function. Our observation that multiple copy genes tend to evolve faster find both support and contradict previous studies. For example, Scannell and Wolfe [43] studied duplicated genes resulting from the whole genome in yeast and found that duplicated genes tend to evolve much faster than single copy genes (i.e. singletons). However, counter examples also exist. Jordan et al. [44] found that duplicated genes in several pairs of species such as human-mouse, fly-mosquito, and yeast-*C. albicans*, on average, evolved slower than singletons. It is not clear what causes the contradictory observations.

### Effect of clustering parameters

Changing the clustering method or parameters will change the composition of functional groups. We tested a number of alternative clustering parameters to determine the effect on our results. For the alternative parameters tested, the ANOVA results showed *p*-values of < 10^-6 ^indicating a strong correlation between *dN*/*dS *and functional groups. For example, using a fixed cluster cutoff height of 0.1 for biological process results in 36 groups, whereas using a cutoff height of 0.001 results in 222 groups. The corresponding *p*-values are < 10^-16 ^and < 10^-6 ^for the analysis of variance for *dN*/*dS *by group. Interestingly, the number of pairs in the "defense response" group does not change and the number of pairs in "protein translation" group changes a little from 53 to 45. This indicates that the results are robust with respect to different clustering parameters.

## Conclusions

This analysis provided empirical evidence for functional bias in molecular evolution rate of genes duplicated by whole genome duplication in *Arabidopsis*. Furthermore, it identified specific functional groups that are likely to have significantly higher or lower molecular evolution rates. For example we found that defense response genes are highly variable while protein translation genes are highly conserved.

Intuitively, these findings are not surprising. Defense response genes should be highly variable to be able to respond to changing environmental conditions, and protein translation genes should be highly conserved since any change would effect all biological functions. Previous studies have shown that the molecular evolution in individual disease defense genes were subject to diversifying selection [45]. It has also been shown that the molecular evolution rate for some ribosomal proteins, which are responsible for protein translation, is slower than that of other known cellular proteins [46]. The fact that these well known patterns emerge from our functional clustering strongly indicate that the method proposed here is a viable one for investigating the relative divergence rates for genes based on their role in the cell, as described by the Gene Ontology. Besides providing independent support to previous findings, our study revealed additional groups of genes such as the ones involved in the endomembrane system in the *α*-duplication event that can be potentially interesting for future empirical studies.

### Future work

Although our results are statistically significant, some additional analysis would define the extent to which function impacts divergence rate in duplicated genes. Further examination of the effect of GC content, protein domains, expression level and gene relocation, would clarify the relation between functional bias and other factors that correlate with divergence rate. Comparison to an outgroup and extending the analysis to another organism such as yeast, will provide supporting evidence and generalize the results to other organisms. It is well known that different functional classes of genes have different levels of expression and that expression has been shown to correlate negatively with nonsynonymous substitutions. A study of how expression level and breadth of expression manifests itself in various functional groups, relative to divergence rate, holds potential for future work.

There are also some refinements to the methods used here that should be considered for future studies. First is to devise a method to combine the three Ontology similarities computed for pairs in GOSim clustering. This should increase the resolution of the groups with respect to their role in the cell and would require only one functional enrichment analysis across all three ontologies, biological process, molecular function and cellular function. Second is to repeat functional clustering with only evidence code filtering, not shared function filtering. This will increase the number of pairs that remain and may find additional emergent functional properties. Finally, consider clustering by GO annotation for individual genes instead of the common annotation for gene pairs. This would produce a more enriched grouping which may provide better insight into the reasons for differences in *dN*/*dS *values.

## Authors' contributions

AW contributed primarily to the clustering and enrichment analysis and RA to the dN/dS and statistical analysis. All authors contributed to the writing of the paper and have read and approved the final manuscript.

## Supplementary Material

Additional file 1 **Supplement 1**. This spreadsheet provides the top 3 enriched terms for all groups and their Bonferroni corrected p-values from each clustering. It also provides information on the group members of each cluster, cluster size, and group height in the hierarchical clustering tree. Click here for file

Additional file 2 **Supplement 2**. Enrichment for Molecular Function: Functional enrichment for molecular function groups 27 (panel A) and 28 (panel B), shows that the groups are highly enriched for structural constituent of ribosome, and enzyme inhibitor activity genes, respectively. For each enrichment figure the left fraction corresponds to the number of genes in the *α*-duplication event with the specific function and the right fraction corresponds to the number of genes with a function in the current group. Click here for file

Additional file 3 **Supplement 3**. Functional enrichment for biological process groups 1, 15, and 24 (panels A, B, and C) shows that the groups are highly enriched for translation, lipid transport, and defense response genes, respectively. Click here for file

Additional file 4 **Supplement 4**. Functional enrichment for cellular component groups 2 (panel A) and 20 (panel B), shows that the groups are highly enriched for cytosolic ribosome, and anchored to membrane genes, respectively. Click here for file

Additional file 5 **Supplement 5**. Chromosomal location of duplicated gene pairs in the analysis. The five chromosomes are shown as black bars with the centromeres depicted as blue dots. Duplicated genes are linked by different colored lines depending on the chromosomes that the duplicates reside on. Only inter-chromosome duplicates are shown. Click here for file

Additional file 6 **Supplement 6**. Analysis of variance between *dN*/*dS *and multiple/single gene pairs. Shows the difference in divergence based on copy number. *Left: *Boxplot showing the median, 25th and 75th percentiles (box), extreme data points excluding outliers (whiskers), and outliers (red crosses). *Right: *The 95% confidence intervals for group means (horizontal lines). Click here for file
